# Spread of Scarlatina and Diphtheria

**Published:** 1881-01

**Authors:** 


### THE SPREAD OF SCARLATINA AND DIPHTHERIA.

THE medical and secular press of the metropolis, contain very full accounts of the prevalence of these fatal diseases in New York and vicinity. The increase in the number of cases reported during the months of December, as shown by the reports of the Sanitary Bureau, is at least ominous. For instance, the week ending November 27, 1880, 110 cases of scarlatina and 122 cases of diphtheria were reported, while for the week ending Dec. 18, 1880, there were reported 151 cases of scarlatina and 167 cases of diphtheria. The mortality from diphtheria has been forty-six per cent., showing the malignant character of the endemic, while over twenty per cent. of the cases of scarlatina have also been fatal.

The Health Physician kindly furnishes us the following monthly statement of the mortality from these diseases for the year 1880, in Buffalo.

| | SCARLATINA. No. of Deaths. | DIPHTHERIA. No. of Deaths. |
|---|---|---|
| January, - - - | 5 | 4 |
| February, - - - - | 11 | 8 |
| March, - - - | 7 | 6 |
| April, - - - - | 5 | 7 |
| May, - - - | 7 | 2 |
| June, - - - - | 4 | 5 |
| July, - - - | 5 | 7 |
| August, - - - - | 10 | 4 |
| September, - - - | 19 | 4 |
| October, - - - - | 23 | 8 |
| November, - - - | 26 | 6 |
| *December, - - - | 24 | 9 |
| Total, - - - | 146 | 70 |

It will be seen that for the first quarter, comprising the months of January, February and March, there were twenty-three deaths from scarlatina and eighteen from diphtheria, while in the last quarter, which includes the months of October, November and December, there were seventy-three deaths from scarlatina and twenty-three from diphtheria. The same authority also states that at least seventy-five per cent. of these deaths occurred in the fifth and sixth wards.

This is certainly an alarming state of the public health, especially if we take into consideration the early period of the season. With the severe months of winter and spring yet to come, it cannot be expected that these diseases will be less prevalent, nor less fatal, unless stringent regulations are enforced for their prevention. With this end in view, the heads of families especially should be warned of the dangers of the disease, and the means necessary to adopt to secure an effectual quarantine. Our public and private school authorities should be authorized to refuse admission to all pupils from families, in which there are or have been cases, for four to six weeks after the subsidence of the active period of the disease. Disinfection is also as important as isolation.

We call attention to these facts at this time, that the profession may be informed of the increasing mortality from these

* To Dec. 25, 1880.

diseases and co-operate with public authorities and with individuals to guard against their further spread.

